# Comparative transcriptomics reveals stage-specific regulatory pathways of ear thickening in *Auricularia polytricha*

**DOI:** 10.1371/journal.pone.0358747

**Published:** 2026-09-25

**Authors:** Yi-Ning Huang, Li-Na Ke, Bin Yuan, Yongwen Lin

**Affiliations:** 1 Zhangzhou Institute of Technology, Zhangzhou, Fujian, China; 2 Zhangzhou Institute of Agricultural Science, Zhangzhou, Fujian, China; Friedrich Schiller University, GERMANY

## Abstract

*Auricularia polytricha* is an economically important edible and medicinal mushroom widely cultivated in China. Ear thickness, as a core commercial trait of this mushroom, directly determines its product quality and economic value. To explore the potential molecular regulatory mechanisms governing ear thickness, we performed comparative transcriptome sequencing on fruiting bodies of the main cultivar ‘Zhang’er 4328’ and its stable thick-ear mutant ‘B2872’ at three key developmental stages: primordium stage (PD), young ear stage (IF), and mature stage (MF). Differentially expressed genes (DEGs) were screened, followed by Gene Ontology (GO) and Kyoto Encyclopedia of Genes and Genomes (KEGG) enrichment analyses. GO enrichment results showed that DEGs were mainly enriched in biological processes associated with metabolic activity, cellular component formation, and catalytic function. KEGG enrichment analysis revealed a distinct stage-specific pattern: DEGs at the PD stage were primarily enriched in energy and substance synthesis pathways (e.g., thiamine metabolism, carbon metabolism); at the IF stage, DEGs were concentrated in pathways related to cell proliferation and structure formation (e.g., amino acid metabolism, nitrogen metabolism); and at the MF stage, DEGs were enriched in pathways involved in protein synthesis and cellular homeostasis maintenance (e.g., ribosome, endoplasmic reticulum protein processing). Our findings indicate that pathways related to energy metabolism, cell proliferation, and protein synthesis provide essential energy and material support for ear thickness in *A. polytricha*. This exploratory study preliminarily fills the research gap in the molecular mechanism underlying ear thickness variation in *A. polytricha* and provides a preliminary theoretical reference for molecular marker-assisted breeding of thick-ear varieties.

## Introduction

*Auricularia polytricha*, belonging to Basidiomycotina, Hymenomycetes, Auriculariales, Auriculariaceae, and the genus *Auricularia*, is a widely cultivated edible and medicinal mushroom in China [1]. Its fruiting bodies are rich in polysaccharides, which exhibit multiple physiological activities including anti-tumor [2], hypolipidemic [3], hepatoprotective [4], neuroprotective [5], renoprotective [6], and antioxidant effects [7,8]. These properties endow *A. polytricha* with broad application prospects in the food and pharmaceutical industries.

Ear thickness is a pivotal commercial trait of *A. polytricha*. High-quality strains with thick ear slices, abundant colloid, and dense white villi demonstrate superior commercial performance after drying. Therefore, breeding thick-ear varieties has become a key direction for the genetic improvement of *A. polytricha*, and clarifying the molecular regulatory mechanism of ear thickness is the core theoretical basis for the directional breeding of this trait.

Currently, studies on edible mushroom development mainly focus on the effects of environmental factors on fruiting body development, flavor formation, and yield improvement [9,10]. Most existing research on *A. polytricha* is limited to variety breeding, cultivation technology optimization, nutrient composition analysis, and genetic relationship evaluation [11]. To date, no systematic studies on the molecular mechanism of stable thick-ear mutants in *A. polytricha* have been reported.

The formation and development of edible mushroom fruiting bodies are regulated by both environmental factors [10](including light, temperature, humidity, ventilation, and pH) and intrinsic molecular mechanisms. At the molecular level, fruiting body growth and development are accompanied by the differential expression of functional genes, and the biological processes regulated by these genes directly determine the yield and quality of fruiting bodies.

With the wide application of high-throughput sequencing technology, RNA sequencing (RNA-Seq) has become an efficient approach to analyze the molecular mechanisms of trait formation in edible mushrooms, promoting breakthroughs in the study of fruiting body development mechanisms. For instance, Du et al. (2019) identified the key regulatory role of the nucleoside diphosphate kinase (NDPK)-encoding gene in the mycelial maturation of *Pleurotus eryngii* through comparative transcriptome analysis [12]. Wang et al. (2018) confirmed that purine metabolism and unsaturated fatty acid metabolism pathways are closely associated with the fruiting body development of *Lentinus edodes* [13]. Recent multi-omic studies in capped basidiomycetes such as *Lentinula edodes* and *Coprinus comatus* have further revealed a conserved stage-specific regulatory pattern, in which energy metabolism, cell wall biosynthesis and ribosome biogenesis sequentially drive primordium differentiation, tissue expansion and mature phenotype fixation [14,15]. RNA-Seq can reveal genome-wide gene expression patterns and mine key functional genes, providing mature and reliable methodological support for analyzing the molecular mechanism of ear thickness in *A. polytricha*.

At present, the molecular regulatory mechanism controlling ear thickness in *A. polytricha* remains largely unclear. The key scientific questions to be addressed in this study are: (1) What stage-specific metabolic and regulatory pathways drive the formation of thick-ear phenotype in A. polytricha? (2) Which core candidate genes play decisive roles in ear thickness at different developmental stages? We hypothesized that the thick-ear phenotype is regulated by a sequential, stage-specific gene network involving energy metabolism, cell proliferation, and protein synthesis pathways. In this study, we used the main cultivated strain ‘Zhang’er 4328’ and its stable thick-ear mutant ‘B2872’ as experimental materials, and performed RNA-Seq on their fruiting bodies at three key developmental stages (PD, IF, MF). We compared DEGs between the two strains at the same developmental stage, mined key genes related to fruiting body differentiation and ear thickness formation, and revealed the stage-specific regulatory pathways of ear thickness. This study not only fills the research gap in the molecular mechanism of ear thickness variation in *A. polytricha* but also provides a reference for molecular marker-assisted breeding of thick-ear varieties and for the study of fruiting body development mechanisms in other edible mushrooms.

## Materials and methods

### Experimental strains

The tested strains were *A. polytricha* ‘Zhang’er 4328’ and its natural thick-ear mutant ‘B2872’. The average ear slice thickness of ‘Zhang’er 4328’ was 0.147 ± 0.008 cm, while that of ‘B2872’ was 0.196 ± 0.011 cm. Both strains were preserved in the Edible Fungi Culture Collection Center of Zhangzhou Institute of Agricultural Sciences.

### Main reagents

The E.Z.N.A.™ Plant RNA Kit was purchased from Annoron (Beijing) Biotechnology Co., Ltd.

Reverse transcription kit and qRT-PCR reagents were purchased from Vazyme Biotech Co., Ltd. (Nanjing, China).

### Sample collection

The cultivation experiment was carried out at Zhangzhou Jinhua Family Farm Co., Ltd. (Zhangzhou, China). ‘Zhang’er 4328’ and ‘B2872’ were inoculated into the same cultivation substrate, which contained 85% wood chips, 12% wheat bran, 3% light calcium carbonate, with a water content of 60%. The substrate was packed into cultivation bags and sterilized by autoclaving at 1.8 kg/cm² for 2.5 h. After inoculation, all bags were incubated at 25–28 °C in darkness until full mycelial colonization, then maintained at 18–23 °C with 85–95% relative humidity, diffuse light and regular ventilation for fruiting body development.

**Fig 1:**
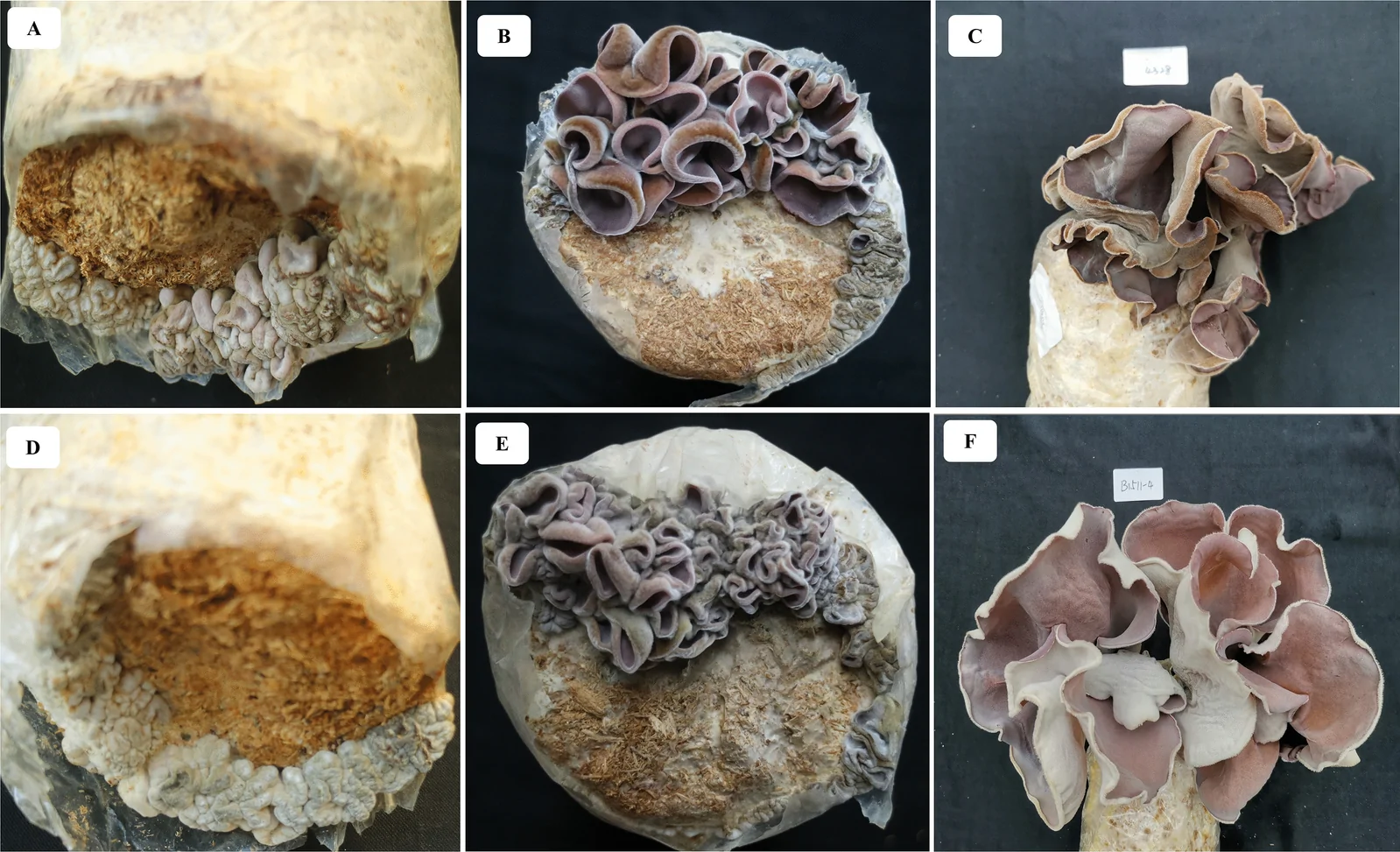
Fruiting bodies of *Auricularia polytricha* at different developmental stages. A: Primordium of wild type ‘Zhang’er 4328’ (CK-PD); B: Initial fruiting of wild type ‘Zhang’er 4328’ (CK-IF); C: Mature fruiting body of wild type ‘Zhang’er 4328’(CK-MF); D: Primordium of thick-ear mutant ‘B2872’(BZ-PD); E: Initial fruiting of mutant thick-ear mutant ‘B2872’(BZ-IF); F: Mature fruiting body of thick-ear mutant ‘B2872’(BZ-MF). All photographs were taken by the authors’ research team.

Fruiting body samples were collected at three key developmental stages: primordium development stage (PD), young ear stage (IF, immature fruiting body), and mature ear stage (MF) (Fig 1). At each stage, 3 ear slices with uniform size and shape were harvested from each strain, mixed evenly, and divided into 3 biological replicates. All samples were immediately snap-frozen in liquid nitrogen, and stored at −80 °C for subsequent RNA extraction. The sample information is shown in Table 1.

**Table 1:** Test samples.

Sample ID	Sample Name	Description
1	CK-PD	Primordium stage of the main cultivated cultivar ‘Zhang’er 4328’
2	CK-IF	Young ear stage of the main cultivated cultivar ‘Zhang’er 4328’
3	CK-MF	Mature stage of the main cultivated cultivar ‘Zhang’er 4328’
4	BZ-PD	Primordium stage of the thick-ear mutant strain ‘B2872’
5	BZ-IF	Young ear stage of the thick-ear mutant strain ‘B2872’
6	BZ-MF	Mature stage of the thick-ear mutant strain ‘B2872’

### Transcriptome analysis

We extracted total RNA from ear slices of different strains using the E.Z.N.A.™ Plant RNA Kit. Transcriptome sequencing was performed on the Illumina high-throughput sequencing platform. Clean reads were obtained by filtering raw data using fastp software with strict thresholds: removing reads containing adapters, reads with N proportion > 10%, reads consisting entirely of adenine (A) bases, and low-quality reads with more than 50% bases of quality value Q ≤ 20.

Clean reads were mapped to the reference genome of *Auricularia polytricha* (its own genome) using HISAT2 software. Gene expression levels were quantified as FPKM (Fragments Per Kilobase of transcript per Million mapped reads) using StringTie software. Differentially expressed genes (DEGs) between wild-type and thick-ear mutant strains were identified using the DESeq2 R package, with screening thresholds of false discovery rate (FDR) < 0.05 and absolute value of log2(fold change) (|log2FC|) > 1.

Gene Ontology (GO) functional enrichment analysis was performed using Blast2GO software and clusterProfiler R package. Kyoto Encyclopedia of Genes and Genomes (KEGG) pathway enrichment analysis was conducted using the clusterProfiler R package. Both GO and KEGG enrichment analyses were performed with a significance threshold of P < 0.05. Sample correlation analysis was calculated based on the Pearson correlation coefficient to evaluate the repeatability of biological replicates.

### qRT-PCR validation

Four DEGs from significantly enriched KEGG pathways were randomly selected for qRT-PCR validation to confirm the reliability of transcriptome data. Total RNA was reverse transcribed into cDNA using a reverse transcription kit. qRT-PCR was performed on a LightCycler 480 real-time PCR system (Roche, Switzerland) using SYBR Green reagent, with A08335 (glyceraldehyde-3-phosphate dehydrogenase, GAPDH) as the internal reference gene. The primer sequences are shown in Table 2. The reaction procedure was as follows: 95 °C for 30 s; 40 cycles of 95 °C for 10 s, 60 °C for 30 s. Each sample was set with 3 biological replicates and 3 technical replicates. The relative expression level of genes was calculated using the 2^−ΔΔCt^ method.

**Table 2:** Primers for qRT-PCR.

Gene ID	Sequence (5’ → 3’)	Gene function
A08335	F: ACGACCGACAAGGCGAAGG R: GCATTGGACACGATGGACAGG	glyceraldehyde-3-phosphate dehydrogenase
A22307	F: ATTACGACGCACCCATCGAC R: GGGACAGTTCCTCCGGTTTT	hypothetical protein EXIGLDRAFT_766705
A17063	F: TTCAAGACGCCGAAGACGAA R: GAAGTAGTACGGCGAGTCGG	glycoside hydrolase family 43 protein
A19784	F: AGACCCAATTCGTCTGCGTT R: CATGGGCGTAATCCAGCTCT	hypothetical protein AURDEDRAFT_187063
A20703	F: CACGAATAACTTGGGCTGCG R: GATCAATGAGCTGCTGCGTG	hypothetical protein SCHCODRAFT_234301

## Results and discussion

### Quality analysis of transcriptome sequencing

A total of 27.58 G, 22.68 G, 26.86 G, 27.65 G, 22.70 G, and 23.50 G of clean data were obtained from BZ-MF, BZ-IF, BZ-PD, CK-MF, CK-IF, and CK-PD samples, respectively. The Q20 value (base recognition accuracy > 99%) of all samples was above 97.0%, and the GC content was between 61.93% and 63.47% (Table 3), indicating that the sequencing data were of high quality and suitable for subsequent analysis.

**Table 3:** Data quality control of tested samples.

Sample	RawData (bp)	CleanData (bp)	AF_Q20(%)	AF_GC(%)
BZ-PD-1	8216525400	8172820857	8011083943 (98.02%)	5186928536 (63.47%)
BZ-PD-2	9161993100	9109505967	8921684668 (97.94%)	5777987941 (63.43%)
BZ-PD-3	9477036600	9429498049	9233084976 (97.92%)	5978473860 (63.40%)
BZ-IF-1	7545501300	7494531400	7301716000 (97.43%)	4719001547 (62.97%)
BZ-IF-2	8273515500	8227239396	7984911928 (97.05%)	5190905959 (63.09%)
BZ-IF-3	6857327700	6813531462	6608670056 (96.99%)	4318477623 (63.38%)
BZ-MF-1	8465154900	8300585294	8057100626 (97.07%)	5180520605 (62.41%)
BZ-MF-2	10721413200	10623653059	10340733118 (97.34%)	6579317690 (61.93%)
BZ-MF-3	8390779200	8335588291	8111183959 (97.31%)	5187864077 (62.24%)
CK-PD-1	6865586400	6826108400	6579915073 (96.39%)	4313809514 (63.20%)
CK-PD-2	7209555000	7171218909	7023025042 (97.93%)	4545248627 (63.38%)
CK-PD-3	9429632100	9379826879	9190200592 (97.98%)	5924033321 (63.16%)
CK-IF-1	7464951900	7414908653	7216363639 (97.32%)	4665681665 (62.92%)
CK-IF-2	8221626000	8165067265	7976211494 (97.69%)	5126320188 (62.78%)
CK-IF-3	7010097300	6962035248	6765201369 (97.17%)	4417094062 (63.45%)
CK-MF-1	9158882400	8961290683	8677087436 (96.83%)	5591614652 (62.40%)
CK-MF-2	8695055100	8527816719	8282835821 (97.13%)	5338988242 (62.61%)
CK-MF-3	9797752200	9644391522	9397032454 (97.44%)	6033795432 (62.56%)

**Fig 2:**
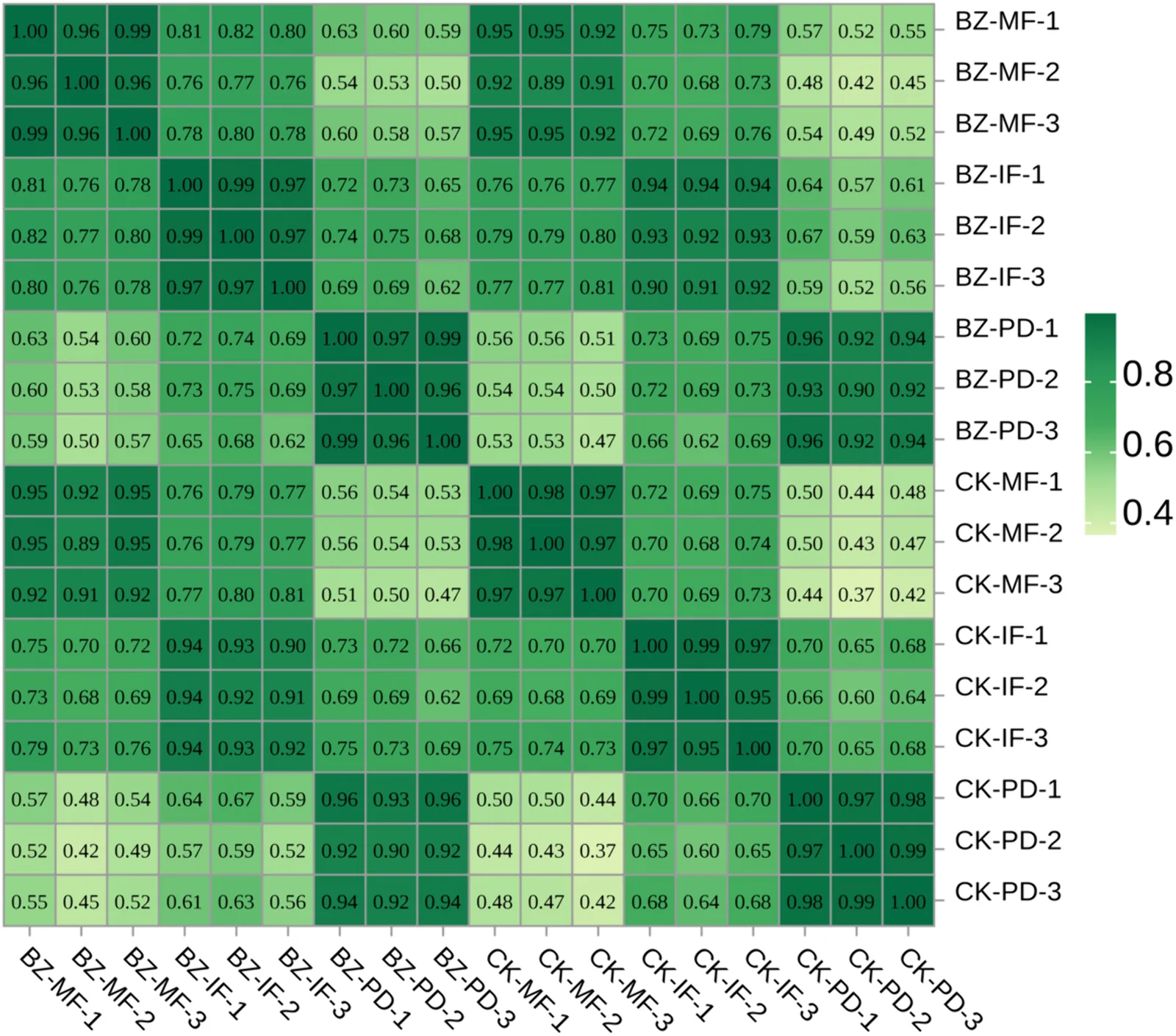
Pearson correlation heatmap of all tested samples. The color gradient represents the correlation coefficient (R²) between samples, with darker color indicating higher correlation. CK-PD: Primordium of wild type; CK-IF: Young ear of wild type; CK-MF: Mature fruiting body of wild type; BZ-PD: Primordium of mutant; BZ-IF: Young ear of mutant; BZ-MF: Mature fruiting body of mutant.

Pearson correlation analysis showed that the correlation coefficient (R²) between biological replicates of the same sample was between 0.9538 and 0.9942 (Fig 2), indicating high repeatability of biological replicates and reliable experimental data.

### Statistics of differentially expressed genes in different strains

**Fig 3:**
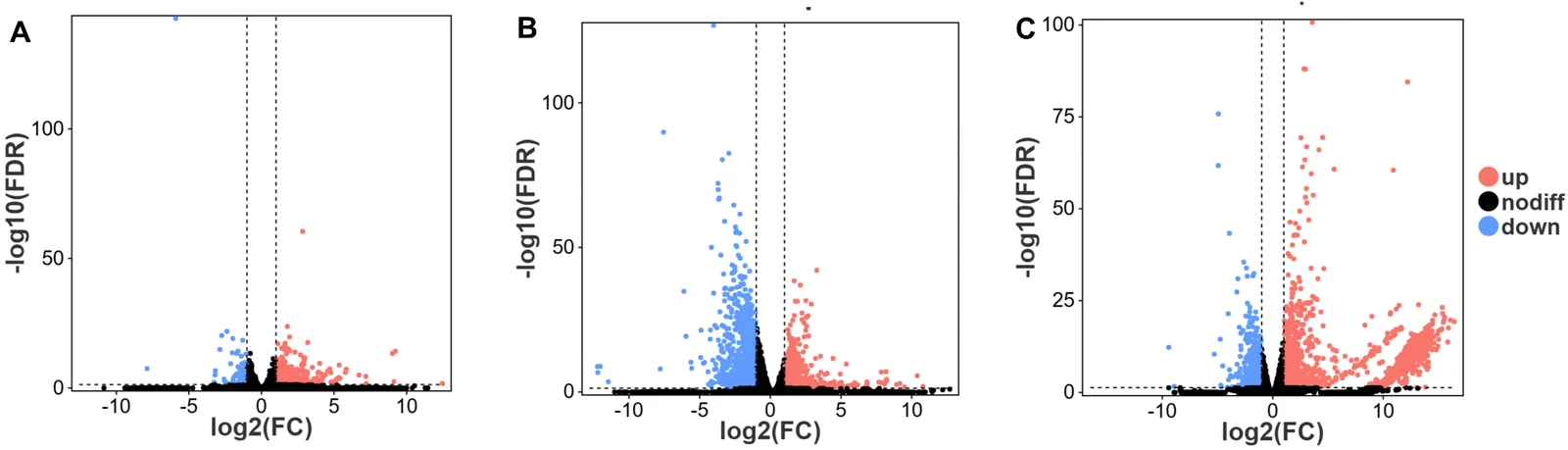
Volcano plots of DEGs between mutant and wild type at different developmental stages. (A) Primordium stage (CK-PD vs BZ-PD); (B) Young ear stage (CK-IF vs BZ-IF); (C) Mature stage (CK-MF vs BZ-MF). Red dots represent significantly up-regulated genes, blue dots represent significantly down-regulated genes, and gray dots represent non-significantly differentially expressed genes. The screening threshold for DEGs was FDR < 0.05 and |log2FoldChange| > 1.

Genes with FDR < 0.05 and |log^2^Fold Change| > 1 were identified as significantly DEGs. The number of DEGs at different developmental stages is shown in Fig 3. Compared with the wild type (CK), 396 DEGs were identified in the mutant (BZ) at PD stage, including 329 up-regulated and 67 down-regulated genes; 1701 DEGs were identified at IF stage, including 805 up-regulated and 896 down-regulated genes; 2623 DEGs were identified at MF stage, including 1976 up-regulated and 647 down-regulated genes. The number of DEGs increased with the development of fruiting bodies, suggesting that the difference in gene expression between the two strains may gradually increased with fruiting body maturation in this study.

### GO functional enrichment analysis of differentially expressed genes in tested strains

**Fig 4:**
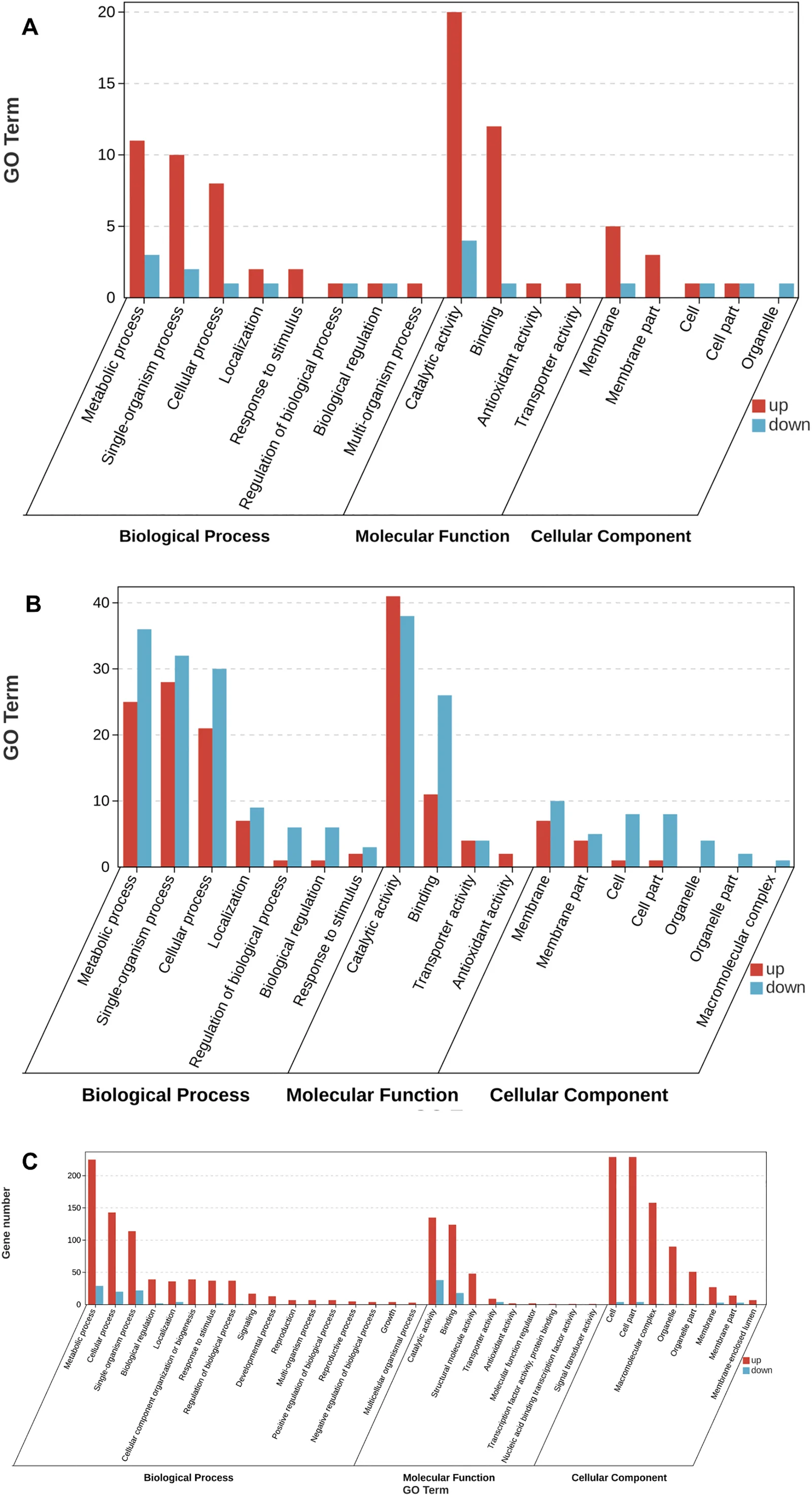
GO functional enrichment analysis of DEGs between mutant and wild type. (A) CK-PD vs BZ-PD (primordium stage); (B) CK-IF vs BZ-IF (young ear stage); (C) CK-MF vs BZ-MF (mature stage). The top 20 significantly enriched GO terms are shown, divided into three categories: Biological Process, Cellular Component, and Molecular Function.

GO functional enrichment analysis was performed on DEGs between the mutant and wild type at three developmental stages (Fig 4). At PD stage, up-regulated DEGs were mainly enriched in biological processes including metabolic process, cellular process, membrane component formation, and catalytic activity, suggesting that the mutant may initiated more active substance metabolism and cell proliferation at the primordium stage. At IF stage, down-regulated DEGs were enriched in metabolic process, cellular process, membrane component, and catalytic activity, while up-regulated DEGs were enriched in transporter activity and antioxidant activity, suggesting that rapid cell proliferation at PD stage slowed down at IF stage, and a large amount of nutrients and growth factors may accumulated to provide a potential material basis for ear slice expansion. At MF stage, up-regulated DEGs were enriched in metabolic process, cellular process, cell part, macromolecular complex, and catalytic activity. Unlike the wild-type strain, in which mature-stage transcription is primarily allocated to normal hymenial differentiation – a conserved developmental program in basidiomycetes – the thick-ear mutant activates an enhanced hyphal growth program on the basis of regular fruiting body maturation. The highly activated pathways related to ear structure synthesis and colloid accumulation promote robust structural protein synthesis, cell wall maturation and gelatinous matrix deposition in hyphal tissues. This enhanced biosynthetic capacity increases overall ear tissue thickness beyond the normal developmental level of the wild type, and ultimately contributes to the final fixation of the significantly thicker ear phenotype.

Overall, GO enrichment results indicated that biological processes related to metabolic activity, cellular component formation, and catalytic function play a key regulatory role in the formation of thick-ear phenotype in *A. polytricha*.

### KEGG enrichment analysis of differentially expressed genes in tested strains

**Fig 5:**
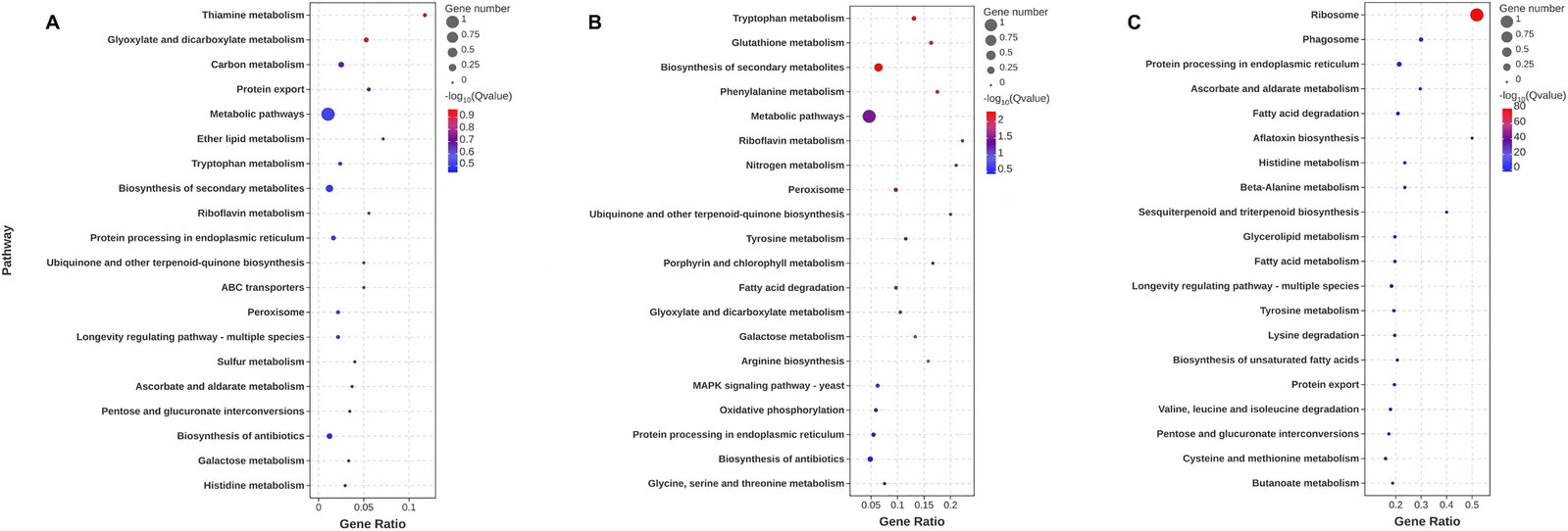
KEGG pathway enrichment analysis of DEGs between mutant and wild type. (A) CK-PD vs BZ-PD (primordium stage); (B) CK-IF vs BZ-IF (young ear stage); (C) CK-MF vs BZ-MF (mature stage). The significantly enriched pathways are shown, with the color gradient representing the P-value of enrichment, and the dot size representing the number of DEGs enriched in the pathway.

KEGG pathway enrichment analysis was performed to reveal the regulatory pathways of ear thickness, and the results showed a significant stage-specific enrichment pattern (Fig 5).

At PD stage, DEGs were significantly enriched in energy and substance synthesis pathways, including thiamine metabolism, glyoxylate and dicarboxylate metabolism, carbon metabolism, pentose and glucuronate interconversions, galactose metabolism, as well as protein export and endoplasmic reticulum protein processing pathways related to cell proliferation and protein synthesis. These pathways are closely related to substance synthesis for primordium differentiation and subsequent ear development.

At IF stage, DEGs were significantly enriched in amino acid and nutrient metabolism pathways, including tryptophan metabolism, glutathione metabolism, phenylalanine metabolism, tyrosine metabolism, arginine biosynthesis, and nitrogen metabolism, as well as peroxisome, ABC transporters, fatty acid degradation, MAPK signaling pathway–yeast, and oxidative phosphorylation pathways related to energy metabolism. These pathways are closely related to the rapid expansion and thickening of *A. polytricha* ear slices at young ear stage.

At MF stage, DEGs were significantly enriched in protein synthesis-related pathways including ribosome and endoplasmic reticulum protein processing, energy metabolism pathways including metabolic pathways and ascorbate and aldarate metabolism, as well as cell structure stability-related pathways including phagosome and cell cycle–yeast. These pathways regulate the synthesis of ear structural proteins, cell wall thickening, and colloid accumulation, and play a potential role in the final fixation of ear thickness at mature stage.

### Identification of key candidate genes regulating ear thickening

Based on the KEGG pathway enrichment results and the differential expression screening criteria (FDR < 0.05, |log2FC| > 1), key candidate genes associated with ear thickness were identified across the three developmental stages. These genes exhibited strict stage-specific expression patterns and were functionally coordinated with the enriched pathways, which may be jointly determined the formation of the thick-ear phenotype (Table 4).

**Table 4:** Key candidate genes regulating ear thickness in *Auricularia polytricha.*

Gene ID	Gene Symbol	Developmental Stage	Related Pathway	log2FC	Putative Biological Function
A019150	AOD1	PD	Carbon metabolism	2.9288	Enhances carbon utilization and ATP supply, promotes primordium cell division
A023336	grp78	PD	Protein export	1.6295	Mediates protein folding and transport, ensures functional maturation of metabolic enzymes
A023349	cf60	PD	Thiamine metabolism	1.1251	Optimizes carbon metabolic flux and energy conversion efficiency
A030677	GAPA	IF	Biosynthesis of secondary metabolites	−7.7905	Redirects metabolic flux to cell-wall polysaccharide and lignin synthesis
A022282	gst2	IF	Glutathione metabolism	4.2905	Enhances antioxidant capacity and maintains redox homeostasis
A023013	PAL	IF	Phenylalanine metabolism	−1.1280	Catalyzes lignin synthesis and strengthens cell-wall mechanical strength
A013132	CYP505A3	IF	Tryptophan metabolism	−2.9125	Regulates growth metabolites and coordinates cell elongation
A025783	RpS27A	MF	Ribosome	15.3124	Core ribosomal protein, massively promotes structural protein synthesis
A010087	RPL3	MF	Ribosome	15.22	Participates in ribosome assembly and improves protein biosynthesis efficiency
A023336	grp78	MF	Protein processing in endoplasmic reticulum	1.6295	Ensures correct folding and quality control of structural proteins

PD, primordium stage; IF, young ear stage; MF, mature stage. Positive log2FC, up-regulated in thick-ear mutant; negative log2FC, down-regulated in thick-ear mutant.

Collectively, these candidate genes constituted a sequential and stage-specific regulatory module for ear thickening in *A. polytricha*. At the primordium stage, genes related to energy supply and protein trafficking were significantly up-regulated to provide sufficient material and energy for the initiation of ear development. During the young ear stage, genes involved in metabolic redistribution, antioxidant defense and cell-wall construction were differentially modulated to drive rapid cell proliferation, cell-wall thickening and tissue expansion. At the mature stage, genes governing ribosome biogenesis and protein processing were overwhelmingly up-regulated to sustain high-level structural protein synthesis, stabilize cell morphology and ultimately fix the ear thickness phenotype. The synergistic action of these genes and pathways may be systematically promotes the development and formation of the thick-ear trait.

### qRT-PCR validation of expression patterns from transcriptome sequencing

**Fig 6:**
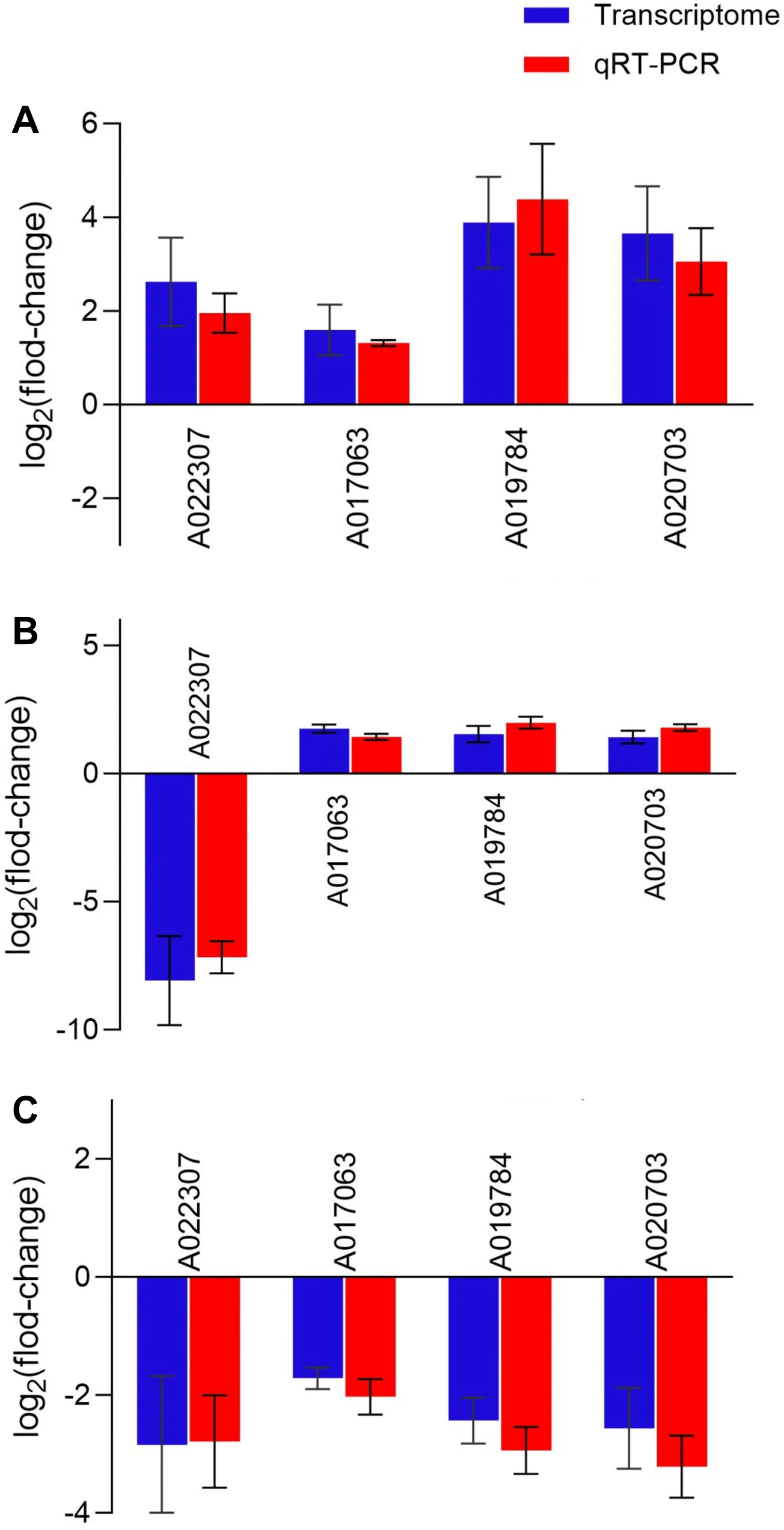
qRT-PCR validation of DEGs. The relative expression levels of four selected DEGs detected by qRT-PCR were consistent with the transcriptome sequencing results. (A) Primordium stage; (B) Young ear stage; (C) Mature stage. Data are shown as mean ± standard deviation of three biological replicates.

The expression trends of the four selected DEGs detected by qRT-PCR were consistent with the transcriptome sequencing results (Fig 6), confirming that the transcriptome data were reliable and accurate.

## Conclusion

In this exploratory study, we performed comparative transcriptome analysis on the fruiting bodies of *A. polytricha* wild type ‘Zhang’er 4328’ and its thick-ear mutant ‘B2872’ at three key developmental stages (PD, IF, MF), and preliminarily screened the potential core DEGs and functional regulatory pathways associated to ear thickness. Notably, the volcano plots exhibited a distinct stage-specific asymmetric distribution: only at the primordium (PD) and mature (MF) stages were significantly more up-regulated genes than down-regulated genes observed in the thick-ear mutant, with the most pronounced global transcriptional activation at the mature stage (1976 up-regulated vs. 647 down-regulated). In contrast, the young ear (IF) stage showed the opposite trend, with more down-regulated genes (896 down-regulated vs. 805 up-regulated). This asymmetric pattern is a genuine biological characteristic rather than an analytical error, representing global transcriptional activation in the mutant. The massive up-regulation of genes was mainly involved in ribosome assembly, structural protein synthesis, cell wall maturation and colloid accumulation. Compared with the wild type characterized by active hymenial development at the mature stage, the thick-ear mutant triggers an additional hyphal growth response: enhanced ribosome biogenesis and protein processing sustain medullary hypha proliferation and deposition of cell wall components and gelatinous matrix, increasing overall ear thickness beyond the normal hymenial layer and providing a direct molecular basis for the mutant’s significantly thickened ear phenotype.

GO enrichment analysis showed that DEGs were significantly enriched in metabolic process, catalytic activity, and cellular component formation, which is consistent with the results of previous studies on fruiting body development of edible mushrooms [13,16]. KEGG enrichment analysis further revealed that the enriched pathways at different developmental stages were closely related to the corresponding biological processes of ear thickening. The primordium stage is the key node for the transition from vegetative growth to reproductive growth in *A. polytricha*, which directly determines fruiting body morphogenesis and ear slice development. We found that DEGs at PD stage were significantly enriched in energy and substance synthesis pathways including thiamine metabolism, carbon metabolism, glyoxylate and dicarboxylate metabolism, and protein export. Carbon metabolism is the core pathway for energy supply and macromolecule synthesis in fungi, which has been confirmed to provide energy and synthetic precursors for mycelial growth of *Hypsizygus marmoreus* and fruiting body morphogenesis of *Stropharia rugosoannulata* and *Flammulina filiformis* [16–18]. Glyoxylate and dicarboxylate metabolism, an important branch of central carbon metabolism, can improve the carbon source utilization efficiency of fungi [19]. Thiamine metabolism regulates the activity of key rate-limiting enzymes in carbon metabolism, thus affecting core metabolic flux and energy conversion efficiency. The protein export pathway can enhance the degradation ability of filamentous fungi to lignocellulose in the cultivation substrate [20]. We found that DEGs at PD stage were significantly enriched in energy and substance synthesis pathways including thiamine metabolism, carbon metabolism, glyoxylate and dicarboxylate metabolism, and protein export. Key genes related to energy supply and protein transport were significantly up-regulated in the thick-ear mutant at this stage, including AOD1 (A0019150), grp78 (A0023336) and cf60 (A0023349). AOD1 enhances carbon utilization efficiency and ATP production as a key rate-limiting enzyme in carbon metabolism; grp78 mediates protein folding and transmembrane transport to ensure the functional maturation of metabolic enzymes; cf60 optimizes carbon metabolic flux and energy conversion efficiency by regulating key enzyme activities. Carbon metabolism is the core pathway for energy supply and macromolecule synthesis in fungi. These pathways work together to provide sufficient ATP and biosynthesis precursors (amino acids, lipids, etc.) for cell division and proliferation at the primordium stage, laying a potential material and energy foundation for subsequent ear thickness. This primordium-stage activation pattern of carbon metabolism and protein secretion is consistent with findings in *Pleurotus ostreatus* and *Lentinula edodes*, two representative capped basidiomycetes, indicating that energy supply initiation is a universal prerequisite for fruiting body morphogenesis [21,22].

The young ear stage is the key period for rapid growth and thickening of *A. polytricha* fruiting bodies, which directly determines the final commercial traits of fruiting bodies. Our results showed that DEGs at IF stage were significantly enriched in amino acid metabolism, nitrogen metabolism, glutathione metabolism, and peroxisome pathways. Previous studies have confirmed that tryptophan, phenylalanine, and tyrosine metabolism can regulate cell wall thickening of *L. edodes* and fruiting body differentiation of *Dictyophora indusiata*, and their product indole acetic acid can drive cell elongation and proliferation [23,24]. Arginine biosynthesis can provide nitrogen sources and protein synthesis precursors for *Agaricus bisporus* fruiting body expansion [25]. Nitrogen metabolism links with carbon metabolism to regulate carbon-nitrogen balance and macromolecule synthesis in fungi [26]. Meanwhile, glutathione metabolism and peroxisome pathways can remove excess reactive oxygen species, maintain cellular redox homeostasis, ensure normal cell proliferation, and enhance energy supply via fatty acid β-oxidation [27,28]. Our results showed that DEGs at IF stage were significantly enriched in amino acid metabolism, nitrogen metabolism, glutathione metabolism, and peroxisome pathways. Key genes involved in metabolic redistribution, antioxidant defense and cell wall synthesis exhibited differential expression patterns: GAPA (A0030677) was extremely down-regulated to redirect metabolic flux toward cell wall structural substance synthesis; gst2 (A0022282) was significantly up-regulated to enhance cellular antioxidant capacity; PAL (A0023013) and CYP505A3 (A0013132) were coordinately regulated to modulate cell wall thickening and cell elongation. These pathways may jointly provide raw materials for protein synthesis, drive cell proliferation and expansion, strengthen cell wall structure, and ensure energy supply and cellular homeostasis, providing core support for ear thickness at young ear stage [29]. Coordinated regulation of amino acid metabolism, phenylpropanoid biosynthesis and cell wall remodeling during the rapid tissue expansion phase has also been widely verified in capped basidiomycetes such as *Flammulina filiformis*, and the associated regulatory module is evolutionarily conserved across Agaricomycetes [30,31].

The mature stage is the final developmental stage of *A. polytricha* fruiting bodies, when ear thickness is basically fixed, and cell differentiation, structure maturation, and nutrient accumulation are highly active. We found that DEGs at MF stage were significantly enriched in ribosome, endoplasmic reticulum protein processing, ascorbate and aldarate metabolism, and phagosome pathways. Ribosome is the core organelle for protein biosynthesis, whose activity is directly related to cell proliferation and differentiation [32], and can synthesize key enzymes and structural proteins for cell wall formation, providing essential protein raw materials for ear cell expansion and cell wall thickening [23,32]. Transcriptomic and proteomic studies on pileus maturation in *L. edodes* and *Agaricus bisporus* both revealed that sustained up-regulation of ribosome and protein processing pathways is a core driver of tissue thickening in capped basidiomycetes at the mature stage [14,33]. The endoplasmic reticulum protein processing pathway is responsible for the folding, modification, and quality control of new proteins, which has been confirmed to regulate protein synthesis and functional maturation during *F. filiformis* fruiting body maturation [16,34]. Ascorbate and aldarate metabolism can remove reactive oxygen species and maintain cell viability under high metabolic levels [35], while the phagosome pathway can remove damaged intracellular components and maintain homeostasis, providing a stable environment for anabolism [36]. We found that DEGs at MF stage were significantly enriched in ribosome, endoplasmic reticulum protein processing, ascorbate and aldarate metabolism, and phagosome pathways. Ribosomal protein genes RpS27A (A0025783) and RPL3 (A0010087) were extremely up-regulated to massively promote structural protein synthesis; grp78 (A0023336) maintained high expression to ensure correct folding and quality control of structural proteins in the endoplasmic reticulum. These pathways may support the vigorous anabolism at mature stage via protein synthesis, functional maturation, and antioxidant protection. Relative to the wild type dominated by normal hymenial differentiation during maturation, the mutant sustains enhanced hyphal proliferation and extracellular matrix accumulation through these activated pathways, which finally contributes to the fixation of increased ear thickness and the formation of superior commercial quality.

At present, most studies on *A. polytricha* focus on cultivation technology optimization, germplasm resource evaluation, and active ingredient extraction, while few studies have reported the molecular regulatory mechanism of fruiting body development, especially ear thickness. In this study, we first performed comparative transcriptome analysis between thick-ear mutant and normal strain of *A. polytricha* at three key developmental stages, and revealed the stage-specific regulatory pathways and candidate genes of ear thickness. This sequential regulatory mode is largely consistent with the conserved fruiting body developmental program documented in diverse capped basidiomycetes, which further supports the reliability and general biological significance of our findings [15,31]. This study fills the research gap on the molecular mechanism of *A. polytricha* ear thickness variation, and provides a theoretical basis for subsequent directional breeding of excellent thick-ear varieties. There are still some limitations in this study: we only screened the core pathways and key candidate genes related to ear thickening based on transcriptome data, and have not performed functional verification of key genes in these pathways. In subsequent studies, we will combine metabolomics, proteomics, and gene function verification to further clarify the specific role of key genes in ear thickness, and deeply reveal the molecular regulatory mechanism of *A. polytricha* fruiting body development.

In conclusion, this study performed the first comparative transcriptomic analysis of wild-type and thick-ear mutant *Auricularia polytricha* across three developmental stages (PD, IF, MF), and identified a stage-specific regulatory network underlying ear thickness. Our results revealed a sequential regulatory pattern: energy supply and protein transport genes (e.g., *AOD1, grp78, cf60*) initiate ear development at the primordium stage; metabolic redistribution, antioxidant and cell wall synthesis genes (e.g., *GAPA, gst2, PAL*) drive rapid ear thickening at the young ear stage; and ribosomal protein and protein processing genes (e.g., *RpS27A, RPL3*) stabilize the thick-ear phenotype at the mature stage. This study preliminarily identifies the putative the core genes and stage-specific pathways governing ear thickness variation in *A. polytricha*, filling the research gap of its molecular regulatory mechanism. The candidate genes identified provide valuable molecular targets for marker-assisted breeding of high-quality thick-ear varieties.
